# Tissue-Specific Distribution of Secondary Metabolites in Rapeseed (*Brassica napus* L.)

**DOI:** 10.1371/journal.pone.0048006

**Published:** 2012-10-25

**Authors:** Jingjing Fang, Michael Reichelt, William Hidalgo, Sara Agnolet, Bernd Schneider

**Affiliations:** Max Planck Institute for Chemical Ecology, Jena, Germany; University of New South Wales, Australia

## Abstract

Four different parts, hypocotyl and radicle (HR), inner cotyledon (IC), outer cotyledon (OC), seed coat and endosperm (SE), were sampled from mature rapeseed (*Brassica napus* L.) by laser microdissection. Subsequently, major secondary metabolites, glucosinolates and sinapine, as well as three minor ones, a cyclic spermidine conjugate and two flavonoids, representing different compound categories, were qualified and quantified in dissected samples by high-performance liquid chromatography with diode array detection and mass spectrometry. No qualitative and quantitative difference of glucosinolates and sinapine was detected in embryo tissues (HR, IC and OC). On the other hand, the three minor compounds were observed to be distributed unevenly in different rapeseed tissues. The hypothetic biological functions of the distribution patterns of different secondary metabolites in rapeseed are discussed.

## Introduction

Seeds, the reproductive organs of plants, generally consist of seed coat, endosperm and embryo. Seed coats protect seeds during dormancy; endosperms normally provide nutrients during germination and, in the initial growth phase of the developing seedling; while embryos, which consist of cotyledons, hypocotyl and radicle, develop into different organs of the seedlings. According to the requirements of different physiological processes, nutrients and other metabolites are distributed and deposited in various seed organs. The embryo – which in the case of rapeseed (*Brassica napus* L.) refers especially to the cotyledons – is a storage site for lipids. In rapeseed, the oil contents reach approximately 50% (w/w) [1], making rape a major oil crop; worldwide it contributes up to 15% of global oil production [2]. Glucosinolates, which account for 3–8% of the rapeseed meal of conventional cultivars and 0.5–1.0% of low-glucosinolate cultivars, may have a depot function for nitrogen, as cyanogenic glucosides do [3]. Phenolic choline esters, mainly sinapate choline esters, are the other major class of secondary metabolites in rapeseed. Sinapine, the choline ester of sinapic acid (sinapate), is the predominant compound of that type, constituting 1–2% (w/w) of the rapeseed meal [4]. Although the sinapine biosynthesis pathway has been well investigated in Brassicaceae plants [5], the biological functions of sinapate choline esters are barely known. Sinapine was thought to be stored in *Raphanus sativus* seeds as a supply of choline, a compound that aids phosphatidylcholine biosynthesis in young seedlings [6]. From a nutritional point of view, the presence of the major secondary metabolites, glucosinolates and sinapates, are unwanted because of their antinutritive properties [1]. However, these compounds are very important for helping plants adapt to their biotic and abiotic environments [7], [8], and in plants different classes of secondary metabolites play specific ecological functions.

The glucosinolate-myrosinase system found in rape and other Brassicales is one of the best-explored plant chemical defense systems against herbivores [9]. Glucosinolate-derived indolics are also involved in antifungal defense [10]. Flavonoids, sinapates and other phenolics have been found in rapeseed and protect plants from ultraviolet-B (UV-B) stress [11]–[13]. Because different classes of secondary metabolites possess individual biological functions, it is reasonable to speculate that diverse secondary metabolites in rapeseed accumulate separately in specific tissues and play different roles in physiological processes or ecological interactions.

A recent study, in which laser microdissection (LMD) was successfully used to harvest specific tissues from developing rapeseed [14], encouraged us to apply LMD to sample different tissues of mature rapeseed and map the distribution of diverse secondary metabolites in the seed tissues. Insights gained from understanding how secondary metabolites are distributed in rapeseed can help us to conceive the biosynthesis and function of these metabolites in the plant.

**Figure 1:**
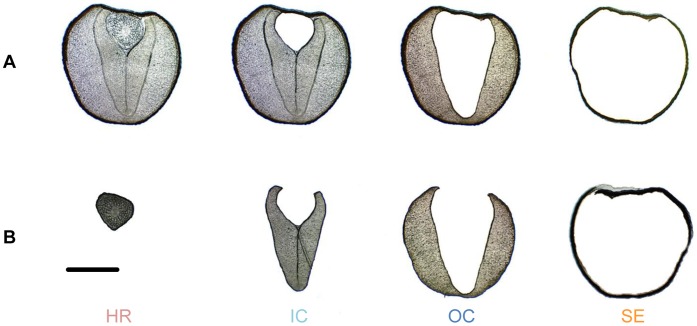
Work flow of laser microdissection of rapeseed. (**A**) Progress of laser microdissection workflow applied to rapeseed. Hypocotyl and radicle (HR), inner cotyledon (IC), outer cotyledon (OC), seed coat and endosperm (SE) were successively dissected from rapeseed. (**B**) Micrographs of dissected tissues. Bar represents 1 mm.

LMD has been successfully used to harvest specific tissues or cells from plant material for transcript and protein analyses [15]–[17], and micro-spatial metabolic profiling studies [18]–[22]. In this study, LMD was used to sample four different parts, namely, hypocotyl and radicle (HR), inner cotyledon (IC), outer cotyledon (OC), seed coat and endosperm (SE) (Figure 1) from mature rapeseed. Secondary metabolites of different classes found in rapeseed cv. “Emerald,” namely glucosinolates, sinapine, a cyclic spermidine conjugate and flavonoids (unpublished data), were quantified in the extracts of dissected tissues by high-performance liquid chromatography - diode array detection and mass spectrometry (HPLC-DAD/MS). Here we report the distribution patterns of the above secondary metabolites in different rapeseed tissues and discuss their potential physiological and ecological relevance.

## Results and Discussion

### Laser Microdissection of Rapeseed

The progress of LMD workflow applied to rapeseed is shown in Figure 1A. Four tissue parts, hypocotyl and radicle (HR), inner cotyledon (IC), outer cotyledon (OC), seed coat and endosperm (SE) (Figure 1B), were successively dissected from rapeseed cryosections and collected for analysis. HR, IC, and OC constitute the rapeseed embryo, and SE is material from the seed hull. The sampling was performed on four individual seeds. The weights of the four parts from each seed are listed in Table 1. The weights include the supporting polyethylene terephthalate (PET) membrane of the frame slide, which was unavoidably cut along with the seed tissues. The dissected materials were prepared for further analysis according to procedures described in the Materials and methods section.

**Table 1:** Weights (mg) of laser microdissected samples obtained from four individual seeds.

Seed	HR	IC	OC	SE
1	0.50	1.19	2.05	0.69
2	0.46	1.11	1.59	0.57
3	0.64	1.00	1.43	0.57
4	0.58	0.98	1.39	0.47

The samples include the supporting polyethylene terephthalate (PET) membrane of frame slides, which was cut together with the seed material. HR: hypocotyl and radicle; IC, inner cotyledon; OC, outer cotyledon; SE, seed coat and endosperm.

### Glucosinolates in Rapeseed

**Figure 2:**
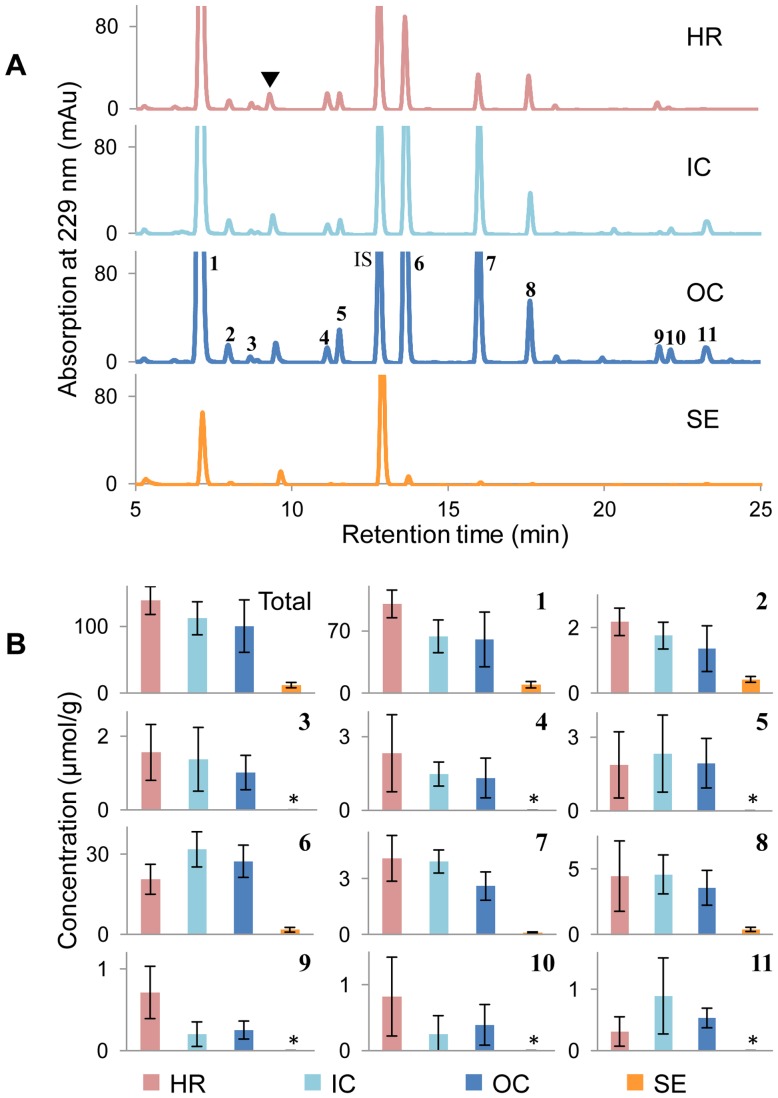
Glucosinolate profiles and distribution in different rapeseed tissues. (**A**) HPLC chromatograms of glucosinolate profiling in laser-microdissected samples from rapeseed detected at 229 nm. ▾ contamination peaks. (**B**) Total glucosinolate concentration and concentrations of individual glucosinolates **1–11** in four dissected samples. HR, hypocotyl and radicle; IC, inner cotyledon; OC, outer cotyledon; and SE, seed coat and endosperm. Each column shows the mean of four replicates with standard error. *means not detectable. Peaks: **1**, progoitrin; **2**, epiprogoitrin; **3**, glucoraphanin; **4**, gluconapoleiferin; **5**, glucoalyssin; **6**, gluconapin; **7**, 4-hydroxyglucobrassicin; **8**, glucobrassicanapin; **9**, glucoerucin; **10**, glucoberteroin; and **11**, gluconasturtiin.

Glucosinolates were determined in their desulfated form by HPLC-DAD/MS at 229 nm. Figure 2A shows chromatograms of the extracts of four seed tissues, HR, IC, OC and SE, dissected from rapeseed. Altogether, 11 desulfated glucosinolates, which have been recently identified in the “Emerald” cultivar of rapeseed (unpublished data), were determined by comparing MS data and retention times with those of references. The concentrations of identified glucosinolates (Figure 2B) from different seed tissues were calculated relative to the internal standard sinalbin. The concentration of glucosinolates in this cultivar is relatively high. Total glucosinolate concentrations in embryo tissues (HR, IC and OC) are higher than 100 µmol/g DW, and they are not statistically different between embryo tissues. Progoitrin (**1**) and gluconapin (**6**) are the predominant glucosinolates in this cultivar as they are in other rapeseed cultivars [23]. In the three embryo parts (HR, IC and OC), glucosinolate profiles are the same, and the individual glucosinolate concentrations are not significantly different. The concentrations of detected glucosinolates in SE samples are significantly lower than those in embryo tissues. Glucosinolates, glucoraphanin (**3**), gluconapoleiferin (**4**), glucoalyssin (**5**), glucoerucin (**9**), glucoberteroin (**10**) and gluconasturtiin (**11**) could not be detected in SE tissues, probably because of the very small amounts of dissected material available for analysis (Table 1), and the SE tissue is dominated by a hard seed coat.

The even distribution of glucosinolates in mature rapeseed embryo tissues (HR, IC and OC) is consistent with the observation that myrosinase is expressed in all embyo tissues of developing rapeseed [24]. Glucosinolates of brassicaceous plants are well-known defense compounds, effective against herbivores and pathogens [25]–[27]. The evenly distributed glucosinolates in HR, IC and OC seem to provide protection for the entire embryo during seed dormancy. Glucosinolate levels decrease during germination of rapeseed [28] and *Arabidopsis thaliana* seeds [29], and the degradation products affect the interaction of plant roots with microorganisms [30]–[36], nematodes [37]–[40], other plants [41]–[43] and animals [39]. These evidences strongly indicate a depot function of glucosinolates in mature rapeseed as precursors of allelochemicals, which help the seedlings to establish the ecosystem in the rhizosphere.

### Sinapine in Rapeseed

**Figure 3:**
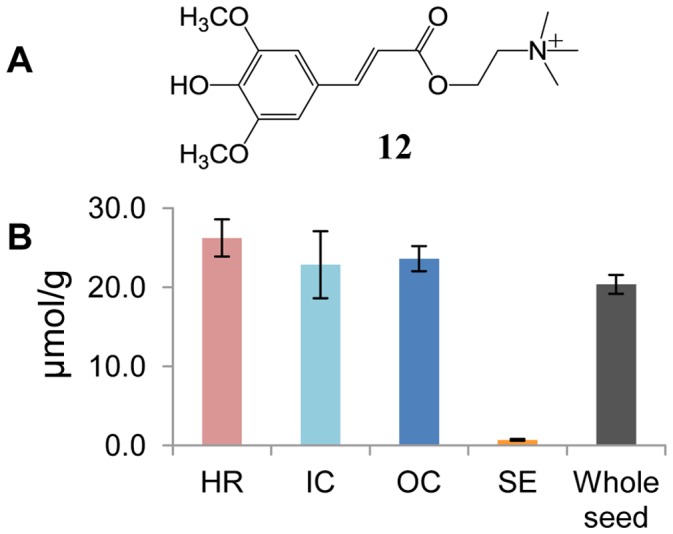
Distribution of sinapine in rapeseed. (**A**) Structure of sinapine (**12**). (**B**) Sinapine concentrations in different rapeseed tissues and whole rapeseed. HR, hypocotyl and radicle; IC, inner cotyledon; OC, outer cotyledon; and SE, seed coat and endosperm. Each column shows the mean of four replicates with standard error.

Sinapine, **12** (Figure 3A), the choline ester of sinapate, represents the dominant phenolic compound in rapeseed. The concentration of sinapine in four tested seeds of the “Emerald” cultivar averaged 20.36 µmol/g. Average sinapine concentrations (Figure 3B) found in three embryo tissues (HR, IC and OC) are close to each other, and all of them are higher than 22 µmol/g. The concentration detected in SE (0.72 µmol/g) is significantly lower than that in the embryo tissues. This finding is in accordance with the reported occurrence of sinapine mainly in rapeseed embryo [44].

Sinapates, which are biosynthesized through the phenylpropanoid pathway, are chemotaxonomic markers of brassicaceous plants [45]. Sinapine is the major compound of that type in mature seeds. During early stages of seedling development sinapine is converted to sinapoylmalate via sinapate and sinapoylglucose [46], [47]. Sinapoylmalate protects plant leaves from UV-B irradiation [12], [48]–[51] and is involved in UV-B-induced defense against fungi in *A. thaliana* leaves [52]. On the other hand, much experimental evidence suggests that the sinapine stored in rapeseed provides a supply of sinapate and choline, both of which serve as important precursors for essential plant components. Sinapine (**12**) degrades into sinapate and choline during early stages of seed germination [6], [53], [54], and the two components are used in later biosynthetic processes [53]. In *Raphanus sativus* seedlings, choline released from sinapine was proven to be processed biosynthetically to phosphatidylcholine [6], and the sinapic acid moiety was hypothesized as the precursor for the biosynthesis of further phenolic compounds, such as flavonoids [53]. Thus, all products released or converted from sinapine during early steps of seed germination (sinapoylglucose, sinapoylmalate, sinapate and choline) play essential physiological and ecological roles for the seedling and plant [5]. The even distribution of sinapine in rapeseed embryo tissue supports its depot function.

### Cyclic Spermidine Conjugates in Rapeseed

**Figure 4:**
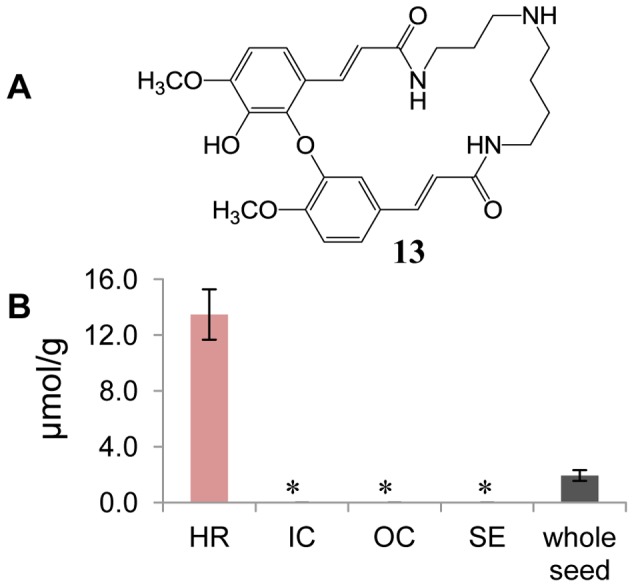
Distribution of the major cyclic spermidine in rapeseed. (**A**) Structure of the major cyclic spermidine conjugate (**13**) identified from rapeseed. (**B**) The concentration of **13** in different tissues and whole rapeseed. HR, hypocotyl and radicle; IC, inner cotyledon; OC, outer cotyledon; and SE, seed coat and endosperm. Each column shows the mean of four replicates with standard error, and *means not detectable.

Cyclic spermidine conjugates in non-glucosinolate (NG) fractions of laser-microdissected rapeseed tissues were detected by HPLC-ESIMS in positive ionization mode (see Materials and methods). The major peak in extracted ion chromatogram (EIC) for ions at *m*/*z* 496.4 ([M+H]^+^) (Figure S1) was identified as the major cyclic spermidine conjugate (**13**) (Figure 4A), based on its molecular mass of 495 Da and comparing the retention time with the compound recently isolated from rapeseed (unpublished data). Based on the same molecular mass in the EIC and the same fragmentation patterns in MS/MS analysis compared to those of the major peak, several minor peaks (Figure S1) were suggested to be isomeric cyclic spermidine conjugates. However, structural details remained unassigned because nuclear magnetic resonance (NMR) data are lacking. The average concentration of compound **13** in the whole rapeseed is 1.94 µmol/g, as calculated from a calibration curve. Interestingly, the cyclic spermidine conjugates were found only in HR, where the average concentration of **13** is as high as 13.48 µmol/g. Compound **13** and minor cyclic spermidines are absent in SE, IC and OC tissues (Figures 4B, S1). No free spermidine was detected in any sample.

Polyamines (PAs) and phenylpropanoid-polyamine conjugates (PPCs) are widely distributed in plants [55], including seeds [56], and play important roles in plant growth, abiotic stress tolerance and defense against insect herbivores [57]–[59]. Compound **13** (Figure 4A) was previously identified as the sole PPC from the same plant material, rapeseed [47], [60]. Nevertheless, this is the first time that the distribution of PPCs in seeds has been directly demonstrated. Our results showed that PPCs in rapeseed accumulate only in HR. This is consistent with the expression of PPC biosynthetic genes in *Arabidopsis* seeds [56]. The same authors also demonstrated that PPCs degrade at an early stage of seed germination [56]. Seeds of an *Arabidopsis* spermidine synthase-deficient double mutant contain a reduced level of spermidine and showed an abnormal phenotype [61]. The results indicated that spermidine, and probably other PAs as well, is essential for seed development in plants. Based on this evidence, PPCs that have accumulated in rapeseed are proposed to be sources of PAs and involved in diverse processes of plant growth and development [57], [58]. Although there is increasing interest on PAs functions in seed germination and seedling growth [62], [63], further experiments are needed to establish the precise roles of PPCs distributed in hypocotyl and/or radicle in rapeseed. Degradation products derived from PPCs also contain phenylpropanoids, which are universal precursors for condensed phenolics in plants.

### Flavonoids in Rapeseed

**Figure 5:**
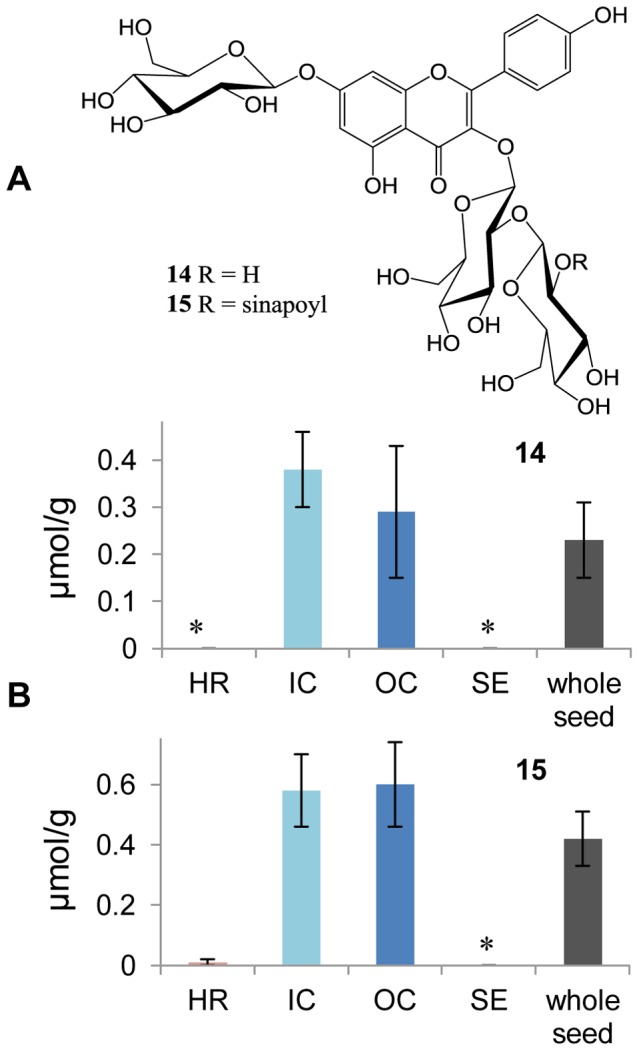
Distribution of the two major flavonoids in rapeseed. (**A**) Structures of two major flavonoids found in rapeseed: **14**, kaempferol-3-*O*-β-D-glucopyranosyl-(1→2)-β-D-glucopyranoside-7-*O*-β-D-glucopyranoside; and **15**, kaempferol-3-*O*-(2-*O*-sinapoyl)-β-D-glucopyranosyl-(1→2)-β-D-glucopyranoside-7-*O*-β-D-glucopyranoside. (**B**) Concentrations of **14** and **15** in different rapeseed tissues and whole rapeseed. HR, hypocotyl and radicle; IC, inner cotyledon; OC, outer cotyledon; and SE, seed coat and endosperm. Each column shows the mean of four replicates with standard error, and *means not detectable.

Two major flavonoids, kaempferol-3-*O*-β-D-glucopyranosyl-(1→2)-β-D-glucopyranoside-7-*O*-β-D-glucopyranoside (**14**) and kaempferol-3-*O*-(2-*O*-sinapoyl)-β-D-glucopyranosyl-(1→2)-β-D-glucopyranoside-7-*O*-β-D-glucopyranoside (**15**) (Figure 5A), are known from the rape cultivar “Emerald” (unpublished data). Using calibration curves, the two flavonoids in dissected rapeseed samples were quantified by HPLC-ESIMS in negative mode. The average concentrations of flavonoids **14** and **15** in the whole seed are 0.23 and 0.42 µmol/g, respectively (Figure 5B). The distribution pattern of flavonoids in different rapeseed tissues is contrary to that of PPCs. Compounds **14** and **15** were mainly detected in cotyledon parts (IC and OC) (Figure S2), where their concentrations are similar. Meanwhile, the two flavonoids are not detectable in SE and almost undetectable in HR (Figure 5B). In fact, a trace of flavonoid **15** was detected in only one of the four HR samples. No kaempferol derivative was detectable in the other three HR samples.

Flavonoids, which constitute an enormously diverse class of phenolic secondary metabolites, are involved in various physiological and ecological processes in plants [64]. A common function of flavonoids is protecting plants from UV-B irradiation [65], which was also demonstrated in rape [66], [67]. Here, the finding of flavonoid accumulation in the primordial tissue of the cotyledons (IC and OC) of mature rapeseed leads to the hypothesis that these compounds are preformed for protecting the chlorophyll and other light-sensitive components from UV-B irradiation in cotyledons emerging during germination. Flavonoids were clearly demonstrated to inhibit root formation [68], [69] by interfering with the transport of auxins from shoot to root [70]–[73]. Our finding that flavonoids are absent in hypocotyl and radicle (HR) fraction is consistent with this physiological phenomenon. Flavonoids also accumulate in seed coats to protect seeds against diverse biotic and abiotic stresses [74]. As in other seeds, proanthocyanidins accumulate in rapeseed coats. Responsible for the seed color, they are normally insoluble [75]. Oligomers and polymers are the probable reason why monomeric flavonoids were not detected in rapeseed hull tissue.

### Tissue-specific Secondary Metabolites Biosynthesis in Rapeseed

**Figure 6:**
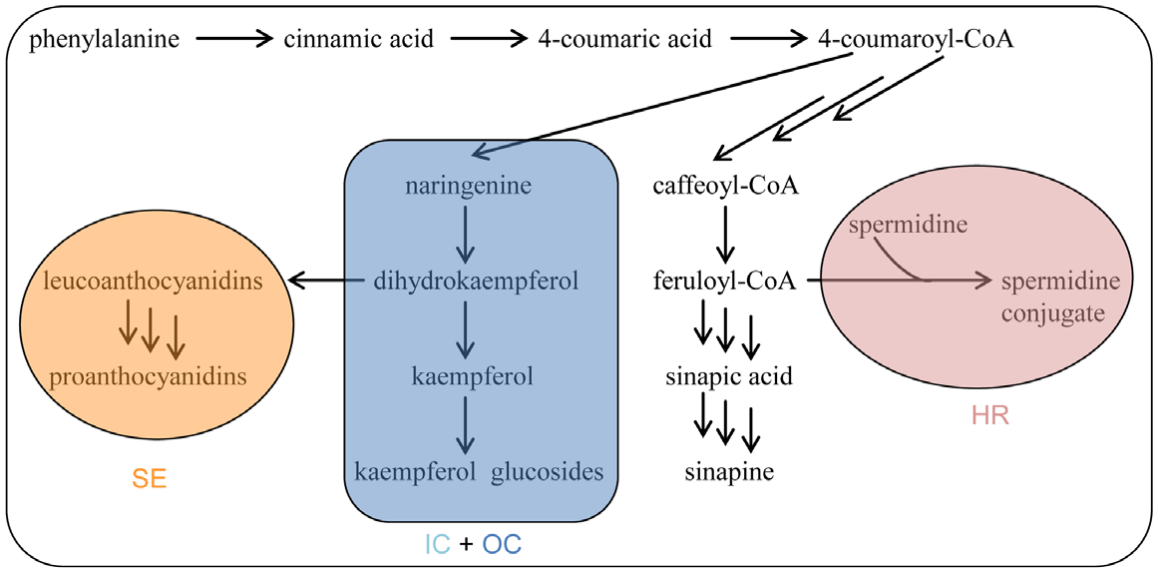
Hypothetical compartementation of phenolics biosynthetic pathways in different rapeseed tissues. HR, hypocotyl and radicle; IC, inner cotyledon; OC, outer cotyledon; and SE, seed coat and endosperm.

The present results and previously reported metabolic profiling data on rapeseed [2], [23], [44], [45], [60], [67], [75], [79] suggest that expression of genes encoding enzymes of secondary metabolites biosynthetic pathways is different among rapeseed tissues. While the glucosinolates are evenly distributed in embryo tissues, and also occur in the seed hull, the phenolics, which all originate from the phenylpropanoid pathway, show tissue-specific distribution patterns disclosing diverse gene expression in rapeseed tissues. The biosynthetic pathways of major phenolics in rapeseed tissues are outlined in Figure 6. Sinapine is synthesized in the entire rapeseed, meanwhile, each tissue pursues its own biosynthetic pathway. Kaempferol glucosides accumulate in cotyledons, suggesting their biosynthesis in this tissue. Another class of flavonoids, the proanthocyanidins are produced in the seed coat [75], [76], the same site as in seeds of other plants [74]. The spermidine conjugate, which is exclusively accumulated in HR, implies that the corresponding biosynthetic pathway occurs only in HR part. The data presented here corroborate the working hypothesis, namely that different classes of secondary metabolites possessing individual biological functions indeed exist in specific tissues in rapeseed.

### Conclusion

Recent studies on the tissue-specific distribution of soluble primary metabolites such as lipids, amino acids, carbohydrates and polymers (starch) demonstrated the feasibility of the LMD-based chemical analysis of rapeseed organs [14]. The major primary metabolites in rapeseed embryo tissues are quantitatively but not qualitatively different, because these components are storage products and are involved in essential life cycles of plant growth and development. Unlike primary components, secondary metabolites help plants adapt to their biotic and abiotic environments [7], [8]. Seed tissues play different roles before and during germination, and develop into individual plant organs after germination. Therefore, secondary metabolites are speculated to accumulate unevenly in different seed tissues. The finding that some of the secondary metabolites detected in this work have different tissue-specific distribution patterns not only solidly supports this hypothesis but also offers the first clue to the biological functions of the secondary metabolites in the mature seed and probably during germination. The knowledge about the specific localization may be used to study the regulation of the biosynthesis and metabolic modification of secondary metabolites. On the other hand, the described sampling methodology, LMD, can be adjusted to facilitate the tissue-specific detection of metabolites, proteins and RNA in other plant materials.

## Materials and Methods

### Plant Material

Rapeseed (winter cultivar “Emerald”) used in this study was purchased from Raps GbR (Langballig, Germany). Entire seeds were used for analysis.

### Laser Microdissection

The basic work flow of LMD and its application to plant tissue has been reported [15], [77]. Mature rapeseed was embedded vertically in Jung tissue freezing medium (Leica Microsystems GmbH, Nussloch, Germany), and immediately frozen in liquid nitrogen. Serial cryosections (60 µm thickness) were prepared at –24°C using a cryostat microtome (Leica CM1850, Bensheim, Germany) and directly mounted on PET-Membrane FrameSlides (MicroDissect GmbH, Herborn, Germany). LMD was performed on the Leica LMD 6000 laser microdissection system (Leica Microsystems GmbH, Wetzlar, Germany) equipped with a nitrogen solid state diode laser of a short pulse duration (355 nm). The cutting settings were as follows: 20×magnification, laser intensity of 128 (the strongest), laser moving speed of 1 (the slowest). The cut materials were collected in the cap of 0.5 ml centrifuge tubes by gravity and then transferred to an HPLC vial. The pictures were taken by a microscope-integrated camera HV-D20P (Hitachi, Tokyo, Japan). Rapeseed was dissected into four parts, HR, IC, OC, and SE (Figure 1), and weights, including the supporting PET membrane of the frame slide, which was unavoidably cut along with the plant tissue, are listed in Table 1.

### Sample Preparation

Generally, each sample was separated into glucosinolate fraction and non-glucosinolate (NG) fraction for further analysis through the procedure adapted from the literature [78]. The four dissected tissue groups (HR, IC, OC, and SE) were extracted separately in an ultrasonic bath for 10 min with 1 ml 80% (v/v) MeOH, which contains 10 µM sinalbin as an internal standard for glucosinolates and 10 µM cinnamic acid choline ester (synthesized according to [79]) as an internal standard for sinapine. The weak anion exchange DEAE Sephadex cartridges (Sigma, Steinheim, Germany), which were conditioned with 800 µl H_2_O and equilibrated with 500 µl 80% (v/v) MeOH before use, were used to separate glucosinolates from the other compounds. Each sample (800 µl extract) was loaded to the cartridge and eluted with 500 µl 80% (v/v) MeOH. Eluate (1300 µl) was collected as an NG fraction and dried in a vacuum centrifuge evaporator Genevac HT-4X (Genevac Ltd, Suffolk, UK). Samples were reconstituted in 200 µl 20% (v/v) MeCN for NG analysis. The DEAE Sephadex cartridges were further eluted by 1 ml H_2_O twice and 500 µl 0.02 M 2-(*N*-morpholino)ethanesulfonic acid (MES) buffer (pH 5.2). Sulfatase (30 µl solution) (Sigma, Steinheim, Germany) was prepared as described in [80] and loaded onto the cartridge. The cartridges were capped, incubated at ambient temperature overnight, and eluted with 500 µl H_2_O for desulfated glucosinolate analysis.

### Identification and Quantification of Glucosinolates

Desulfated glucosinolates were identified with HPLC-DAD/MS by comparing their mass spectrometric data and retention times with those of references [81]. The compounds were quantified based on an internal standard with DAD. HPLC was conducted on an Agilent series HP1100 (binary pump G1312A, autosampler G1367A, diode array detector G1315A; Agilent Technologies, Waldbronn, Germany). Chromatographic separation was performed on a LiChrospher RP18 column (5 µm, 250×4.6 mm, Merck, Darmstadt, Germany) with a guard column (5 µm, 4×4 mm) using a linear binary gradient of H_2_O (solvent A) containing 0.2% (v/v) formic acid (FA) and MeCN (solvent B), with a flow rate of 1.0 ml min^−1^ at 25°C as follows: 0 min: 1.5% B, 1 min: 1.5% B, 6 min: 5% B, 8 min: 7% B, 18 min: 21% B, 23 min: 29% B, 23.1 min: 100% B, 24 min: 100% B, 24.1 min: 1.5% B, and 28 min: 1.5% B. The injection volume was 50 µl. The absorption of HPLC eluate was monitored by DAD at 229 nm.

### Identification and Quantification of Phenolics in the NG Fractions

HPLC-ESIMS was applied to quantify phenolics in laser-microdissected samples in NG fractions. The chromatographic separation was performed on a Nucleodur Sphinx RP column (5 µm, 250×4.6 mm; Macherey-Nagel GmbH, Düren, Germany) using the above-mentioned separation conditions (HPLC system, flow rate, temperature, and eluent) except a linear gradient, which was as follows: 0 min: 10% B, 20 min: 30% B, 25 min: 70% B, 25.1 min: 100% B, 28 min: 100% B, 28.1 min: 10% and 32 min: 10% B. The injection volume was 10 µl. Electrospray ionization mass spectra of HPLC eluate were monitored on an Esquire 6000 ion trap mass spectrometer (Bruker Daltonics, Bremen, Germany). Both positive and negative modes were used in the range *m/z* 150–1200 with skimmer voltage +/−40 V, capillary exit voltage +/−150 V, capillary voltage −/+4000 V, nebulizer pressure 35 psi, drying gas 10 L min^−1^, and gas temperature 350°C. Phenolics were identified based on their MS data and comparing the chromatographic retention times to those of compounds reported for rapeseed of cv. “Emerald” (unpublished data). The concentration of sinapine was calculated relative to the internal standard cinnamic acid choline ester in positive mode. The cyclic spermidine conjugate and two flavonoids were quantified using calibration curves in positive and negative modes, respectively.

### Data Analysis

The experiments were performed in four replicates. Data are reported as means ± standard deviation (SD). Analyses of variance and significant differences among means were tested by one-way ANOVA using SPSS Statistics 17.0. The least significant difference at P = 0.05 level was calculated.

## Supporting Information

Figure S1 **Extracted ion chromatograms for the cyclic spermidine in different rapeseed tissues.** Extracted ion chromatograms (EIC) for ions at *m*/*z* 496.4±0.5 measured in positive ionization mode of samples from different laser-microdissected rapeseed tissues. **13**: Major cyclic spermidine conjugate (for structure, see Figure 4**A**). ▾ major cyclic spermidine conjugate (**13**) peak.HR, hypocotyl and radicle; IC, inner cotyledon; OC, outer cotyledon; and SE, seed coat and endosperm.
(TIF)

Figure S2 **Extracted ion chromatograms for the two major flavonoids in different rapeseed tissues.** Extracted ion chromatograms (EIC) of samples from different rapeseed tissues measured in negative ionization mode for (**A**) ions at *m*/*z* 771.4±0.5 of flavonoid **14**; and (**B**) ions at *m*/*z* 977.5±0.5 of flavonoid **15**. For structures, see Figure 5A. HR, hypocotyl and radicle; IC, inner cotyledon; OC, outer cotyledon; and SE, seed coat and endosperm. ▾ peaks of flavonoid **14** in (**A**) and peaks of flavonoid **15** in (**B**).
(TIF)
