# Supplementary figures and images for: Tissue-Specific Distribution of Secondary Metabolites in Rapeseed (Brassica napus L.)
Source: PLoS One. 2012 Oct 25;7(10):e48006. doi: 10.1371/journal.pone.0048006 (PMC3485038; doi:10.1371/journal.pone.0048006)

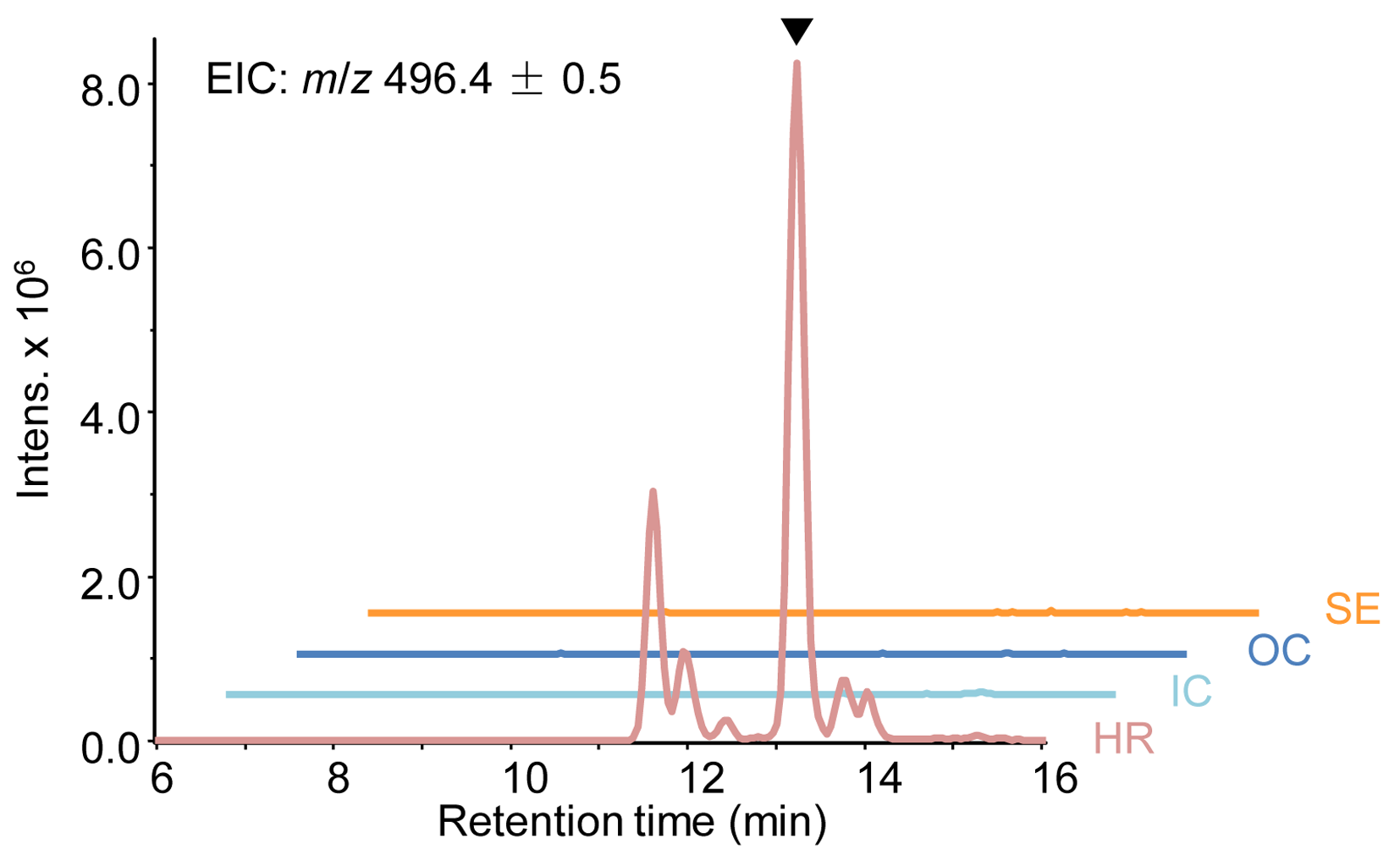

Supplement: Figure S1 — Extracted ion chromatograms for the cyclic spermidine in different rapeseed tissues. Extracted ion chromatograms (EIC) for ions at m/z 496.4±0.5 measured in positive ionization mode of samples from different laser-microdissected rapeseed tissues. 13: Major cyclic spermidine conjugate (for structure, see Figure 4A ). ▾ major cyclic spermidine conjugate (13) peak.HR, hypocotyl and radicle; IC, inner cotyledon; OC, outer cotyledon; and SE, seed coat and endosperm. (TIF) [file pone.0048006.s001.tif]

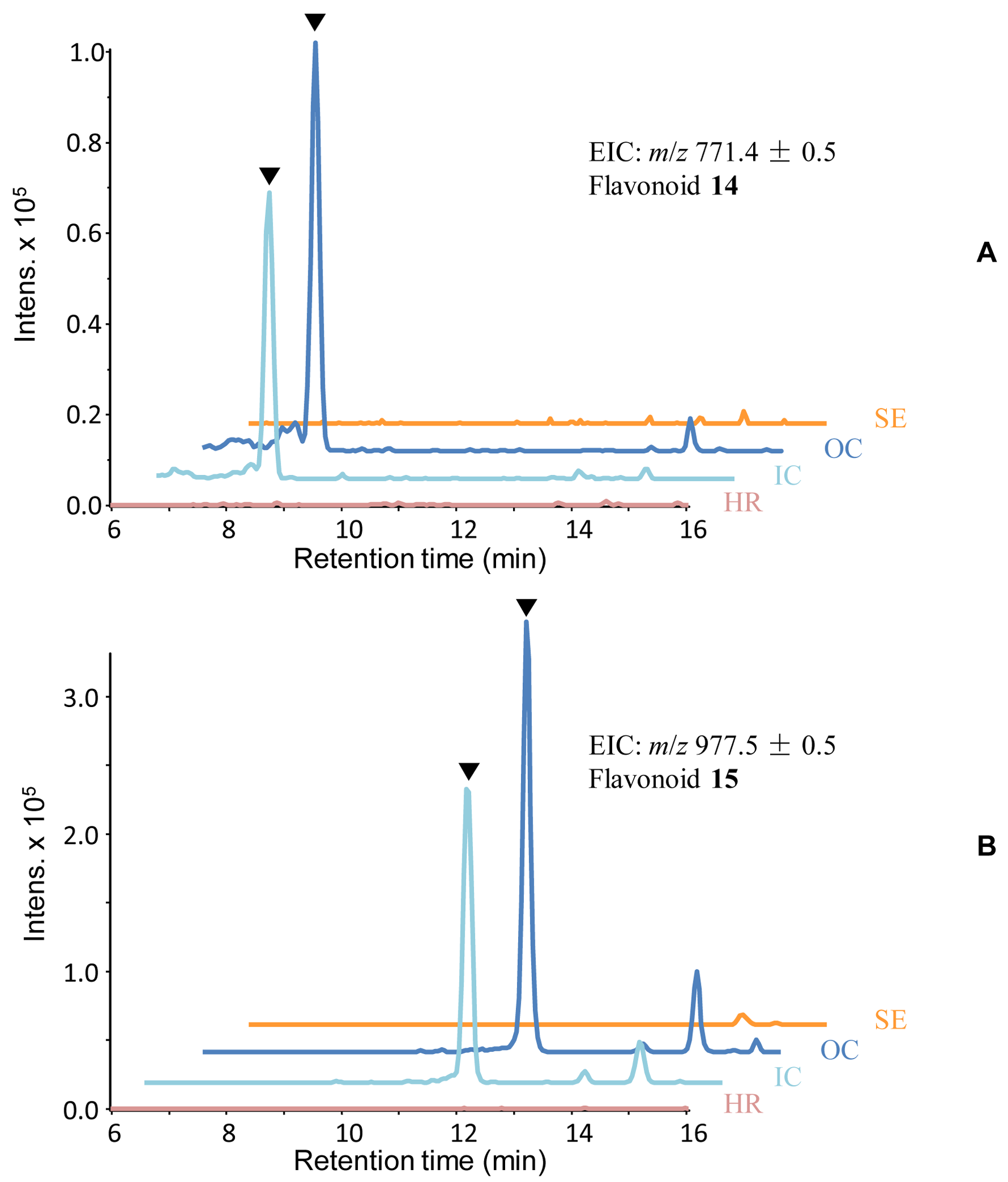

Supplement: Figure S2 — Extracted ion chromatograms for the two major flavonoids in different rapeseed tissues. Extracted ion chromatograms (EIC) of samples from different rapeseed tissues measured in negative ionization mode for (A) ions at m/z 771.4±0.5 of flavonoid 14; and (B) ions at m/z 977.5±0.5 of flavonoid 15. For structures, see Figure 5A. HR, hypocotyl and radicle; IC, inner cotyledon; OC, outer cotyledon; and SE, seed coat and endosperm. ▾ peaks of flavonoid 14 in (A) and peaks of flavonoid 15 in (B). (TIF) [file pone.0048006.s002.tif]
